# PB.42: Arbitration of round masses: understanding the variability of recall rates

**DOI:** 10.1186/bcr3542

**Published:** 2013-11-08

**Authors:** AL Wharton, CG Taylor, JC Cooke

**Affiliations:** 1 Jarvis Breast Screening Centre, Guildford, UK

## Introduction

It is important to keep recall rates to a minimum. At the Jarvis Centre daily arbitration is performed by two experienced film readers, a regular pair each weekday. Observation that different arbitration pairs have varying thresholds for recalling round masses prompted this audit.

## Methods

Thirty-six digital cases with round masses were selected during arbitration over a 1-month period. All cases were anonymised and re-arbitrated by six pairs of arbitrators who usually work together. The pairs included at least one film reader with over 10 years experience. Specific mammographic features and the final audit recall decision were recorded.

## Results

A total 17/36 (47%) cases were recalled at the original arbitration. Arbitration pairs had different audit recall rates ranging from 39 to 81%. All arbitrators agreed to either rescreen or recall in 1/3 of cases. Results showed there was variation in both perception of mammographic features present and the importance given to other factors such as age, multiplicity, lesion location and density; affecting recall even in the presence of benign mammographic features.

## Conclusion

There is considerable variation in opinion between arbitration pairs due to the complex nature of interpreting which mammographic features are present and balancing these against other factors such as age, multiplicity, lesion location, and density. It is useful to reflect on the steps in the decision-making process to understand the science behind the art in an attempt to achieve more consistency between pairs and keep recall rates down.

